# The Introductories

**Published:** 1902-10-11

**Authors:** 


### The Hospital.

A Journal of

The Medical Sciences and Hospital Administration.

Vol. XXXIII.—No. 837.] Edited by Sir Henry Burdett, K.C.B., and Solomon C. Smith, M.D., M.R.C.P. [October 11, 1902.

### The Introductories.

Among the introductory lectures delivered at metropolitan and provincial schools of medicine on the 1st instant, there was, perhaps, none more thoroughly suggestive, or calling more distinctly for reflection, than that given by Sir Dyce Duckworth at Owens College. Sir Dyce, with characteristic courage and candour, declared his conviction that no one who studied the matter with an open mind could believe that the medical profession held its legitimate place in public estimation, and added the expression of his belief that the estimate now formed of it was lower than that of half a century ago. He declared it to be certain that the professional knowledge and ability of the great mass of practitioners had increased enormously in recent years, and that the present generation is better served, in all its ills and sufferings, than any that has preceded it; but added that the degree in which this was appreciated, either by departments of the State or by the public, was little adequate to the facts of the case. If this be a correct description of the existing circumstances of the profession, it is unquestionably one with which its members should not suffer themselves to rest content. Whatever may be the case with the individual doctor, whose power in the sick-room will always depend more upon his personal qualities than even upon the opinion which may be formed of his skill, the power of the profession to influence public conduct must be mainly dependent upon the estimate which is formed of it as a whole. If this estimate be unduly depreciatory, the influence of the profession upon events will be correspondingly impaired. In such a case it becomes a duty to investigate the grounds upon which the prevailing opinion is based, and to see how far, or in what manner, they may be corrected, and to what extent medical men are themselves responsible for them. The profession, as a whole, should bethink itself of the motto of the house of Courtenay, and should say not only "*Ubi lapsus,*" but "*Quid feci*"?

It may be maintained, with a considerable degree of probability, that the condition indicated by Sir Dyce Duckworth is due, in some measure at least, to the recent growth of other professions, which serve to alter the relative position of medicine in the community. When the "three black graces" stood alone, and when Law, Physic, and Divinity were the only callings recognised as conferring the "professional status," the first and last of the three took easy precedence of physic by reason of the higher dignities to which they led. The curate might be Primate of All England, the "devil" of the successful advocate might be Lord Chancellor; while the highest prospects even of the Court physician or surgeon did not soar beyond the modest elevation of a baronetcy. Within the recollections of men not yet old, the triumvirate of "professions" has been largely increased. Literature, art, engineering in its several branches, the stage, are only a few of the callings which must now be added to the list; while, looking at the question in its social aspects, we may see that the Vespasianic *non olet* has come to be applied by the aristocracy to the wealth which has been gained, not only by trade, but even by delinquency. In many of the new professions, moreover, there is an appearance of greater certainty of result, of greater command over the elements of the problems to be dealt with, than has hitherto existed in medicine. The calculations of the engineer or of the electrician rest upon the ascertained and measurable qualities of inert matter, and hence are not liable to be disturbed by unknown or incalculable factors; still less are the followers of those callings condemned to be defeated, sooner or later, by the death of the machine on which they are engaged. In many of the new professions, moreover—especially, perhaps, in engineering—it is generally possible to overcome inferiority of skill by lavishness of expenditure, and so to obtain, even if at a cost the extravagance of which is known only to the initiated, a result which will be hailed by the uninitiated as a triumph of genius. Hence it has befallen that the public, devoid of any real comprehension of the facts of the case, has come to regard certainty and success as the chief criteria of professional ability, and to undervalue the more modest, if more truthful, claims which can be correctly advanced on behalf of the professors of the healing art. Underlying this erroneous estimate, moreover, and to a great extent responsible for it, is that ignorance of science which, in view of its growth, and of its daily increasing applicability to the events of life, is becoming more and more a national disgrace, and threatens to become a source of national disaster. The average man who is supposed to be educated, who can even read a French novel, or quote a line of Horace, has seldom been brought into contact with demonstration in the scientific sense, and hardly realises that there is such a thing as truth, capable of being distinguished from opinion. Still less does he realise the character of the uncertainties of science, or of the mental attitude of the philosopher in relation to them. He does not see that the difficulties of medicine are due to the nature of the occurrences with which it deals, and that these difficulties are only to be removed by research, and not by silly scoffing at men who "can render a reason." A system of national education in harmony with the necessities of the day, if we could ever attain to it, might be expected in time to bring about a change. In the meanwhile, the case is one in which some degree of self-examination may rightly be demanded from the profession itself. Are we ourselves doing our best, striving to guide our footsteps by such light as we possess, and earnestly seeking for more ?

